# Real-world treatment patterns and outcomes among individuals receiving first-line pembrolizumab therapy for recurrent/metastatic head and neck squamous cell carcinoma

**DOI:** 10.3389/fonc.2023.1160144

**Published:** 2023-05-22

**Authors:** Christopher M. Black, Glenn J. Hanna, Liya Wang, Karthik Ramakrishnan, Daisuke Goto, Vladimir Turzhitsky, Gleicy M. Hair

**Affiliations:** ^1^ Center for Observational and Real-World Evidence (CORE), Merck & Co., Inc., Rahway, NJ, United States; ^2^ Center for Head & Neck Oncology, Dana-Farber Cancer Institute, Boston, MA, United States

**Keywords:** head and neck squamous cell carcinoma, antineoplastic agents, immunological, antibodies, Kaplan-Meier estimate, patient outcomes, real-world observational study, treatment patterns

## Abstract

**Background:**

Pembrolizumab, a PD-1 immune checkpoint inhibitor, is approved as first-line (1L) treatment for recurrent or metastatic head and neck squamous cell carcinoma (R/M HNSCC) as monotherapy or in combination with platinum and 5-fluorouracil chemotherapy. Limited data exist on the use of these regimens in real-world settings.

**Objective:**

Our primary objectives were to describe baseline characteristics and real-world overall survival (rwOS), time on treatment (rwToT), and time to next treatment (rwTTNT) among individuals with R/M HNSCC receiving approved 1L pembrolizumab therapies. We also aimed to identify baseline factors associated with choice of 1L pembrolizumab therapy and with rwOS.

**Methods:**

This was a retrospective cohort study of adults with R/M HNSCC receiving 1L pembrolizumab monotherapy or pembrolizumab plus chemotherapy. We used Kaplan-Meier analyses to assess real-world outcomes, logistic regression modeling to identify factors associated with choice of 1L pembrolizumab therapy, and Cox proportional hazards models to identify factors associated with rwOS.

**Results:**

The study population included 431 individuals receiving 1L pembrolizumab monotherapy and 215 receiving 1L pembrolizumab plus chemotherapy. The use of 1L pembrolizumab monotherapy was associated with higher baseline combined positive score for PD-L1 expression, older age, higher Eastern Cooperative Oncology Group performance status (ECOG PS), laryngeal tumor site, and human papillomavirus (HPV)-positive tumor status. The pembrolizumab monotherapy group had a median (95% CI) rwOS of 12.1 (9.2–15.1) months, rwToT of 4.2 (3.5–4.6) months, and rwTTNT of 6.5 (5.4–7.4) months. Among this group, HPV-positive tumor status and lower ECOG PS were associated with longer rwOS, and oral cavity tumor site with shorter rwOS. The pembrolizumab plus chemotherapy cohort had a median (95% CI) rwOS of 11.9 (9.0–16.0) months, rwToT of 4.9 (3.8–5.6) months, and rwTTNT of 6.6 (5.8–8.3) months. In this group, HPV-positive tumor status was associated with longer rwOS.

**Conclusions:**

This study adds to clinical trial data by summarizing real-world treatment outcomes with 1L pembrolizumab-containing therapies in a more heterogeneous population. Overall survival outcomes in both treatment groups were similar to those observed in the registration clinical trial. These findings support the use of pembrolizumab as standard of care for R/M HNSCC.

## Introduction

Tumors that occur in the head and neck collectively account for ~4% of all cancers in the US (1). More than 90% of these cancers are classed as head and neck squamous cell carcinomas (HNSCCs), which can occur at multiple sites (2). Risk factors for HNSCC include male sex, smoking, alcohol consumption, and infection with oncogenic strains of the human papillomavirus (HPV) (3). When diagnosed at an early stage the 5-year survival rate for HNSCC is 70–90% (4). However, around half of HNSCCs have evidence of regional lymph node involvement at the time of diagnosis (i.e., locoregionally advanced HNSCC); this status is associated with an 18–51% rate of local or regional recurrence and a 13–20% rate of distant metastasis within 5 years (4–7). Prior to 2019, individuals with recurrent or metastatic (R/M) HNSCC had a poor prognosis, with a median overall survival of 6–10 months (4, 8, 9).

In June 2019, programmed death-1 (PD-1) immune checkpoint inhibitor pembrolizumab (KEYTRUDA^®^) was approved by the US Food and Drug Administration (FDA) as first-line (1L) treatment for R/M HNSCC (10). The FDA’s approval was based on the findings of the KEYNOTE-048 clinical trial (ClinicalTrails.gov NCT02358031), which showed a statistically significant improvement in overall survival (OS) and a more durable anti-tumor response for pembrolizumab monotherapy or pembrolizumab in combination with platinum and 5-fluorouracil (5-FU)—henceforth referred to as ‘pembrolizumab plus chemotherapy’—compared to the previous standard of care, cetuximab plus platinum and 5-fluorouracil (known as the EXTREME regimen) (11). At final analysis, favorable OS results were observed for pembrolizumab monotherapy in the programmed death-ligand 1 (PD-L1) combined positive score (CPS) ≥1 population (median OS 12.3 months versus 10.3 months; hazard ratio [HR] 0.74 [95% CI 0.61–0.90]) and for pembrolizumab plus chemotherapy (median OS 13.0 months versus 10.7 months; HR 0.72 [95% CI 0.60–0.87]) in the overall R/M HNSCC population compared to the EXTREME regimen. Furthermore, pembrolizumab monotherapy was a better-tolerated 1L treatment than the EXTREME regimen, and has since been found to be dominant or cost-effective compared with other common treatment regimens (11, 12). A 4-year follow-up study found continued survival benefits with 1L pembrolizumab-containing therapies compared to the EXTREME regimen (13). Consistent with the FDA approval, the National Comprehensive Cancer Network’s 2022 Clinical Practice Guidelines for Head and Neck Cancers recommend pembrolizumab monotherapy for tumors that express PD-L1 with CPS ≥1 (category 1) and in combination with platinum and 5-FU chemotherapy (category 1) as the preferred 1L treatment option for R/M HNSCC (14).

There is limited information on the real-world use of FDA-approved 1L pembrolizumab-containing therapies for R/M HNSCC. The primary objectives of this study were to use real-world data to describe the baseline demographic and clinical characteristics of individuals receiving 1L pembrolizumab monotherapy and pembrolizumab plus chemotherapy for R/M HNSCC and to assess their OS and treatment outcomes. The secondary objectives were to identify baseline individual factors associated with choice of 1L pembrolizumab treatment and with rwOS.

## Methods

### Study design

This retrospective observational cohort study selected individuals with R/M HNSCC initiating 1L pembrolizumab monotherapy or pembrolizumab plus chemotherapy between July 1, 2019 and December 31, 2021, following FDA approval of these regimens on June 10, 2019. The index date was defined as the date of initiation of 1L pembrolizumab monotherapy or pembrolizumab plus chemotherapy. Demographic and clinical characteristics were summarized during the baseline period using all available records from the database inception date (January 1, 2011) up to the index date plus 30 days. All study outcomes were assessed during the follow-up period from index date until data cut-off on June 30, 2022, thus enabling a potential follow-up period of ≥6 months. Individuals were censored at death (for all outcomes except OS) or lost follow-up (all outcomes).

### Study population

The study population was drawn from the nationwide Flatiron Health electronic health record (EHR)-derived de-identified database, which includes data from ~280 cancer clinics (~800 sites of care) (15, 16). The database represents 17% of incident cancer cases in the US and is the largest national source of real-world longitudinal oncology data. Structured and unstructured data (including lab test and biomarker values and physician notes) are extracted from EHRs, processed, and included in a single database, which is refreshed monthly.

Individuals 18 years of age or older from the Flatiron Health advanced HNSCC (aHNSCC) database (17) with a diagnosis of advanced head and neck cancer involving the hypopharynx, larynx, oropharynx, or oral cavity were potentially eligible for inclusion in the study. Individuals with HNSCC tumors at other sites were excluded. Head and neck cancers were identified using relevant International Classification of Diseases codes (**Supplementary Table 1**). Individuals were classified as having aHNSCC if they had Stage IVc disease at initial diagnosis; had received locoregional disease management with non-curative intent; or had distant disease recurrence. Each diagnosis of aHNSCC, and the associated date, was confirmed manually by trained chart abstractors with input from clinical oncologists. Additional study inclusion criteria were ≥2 documented clinical visits on or after January 1, 2011, and initiation of 1L systemic treatment for R/M HNSCC. Individuals were excluded if they had a record of platinum chemotherapy within the 6-month period prior to initiation of 1L treatment or if they received a clinical trial drug during the study period.

All systemic therapies were defined according to line of treatment (LOT). The start of 1L therapy was defined as the first episode of an eligible therapy that was given after or ≤14 days before the aHNSCC diagnosis date and after the start of structured activity (i.e., record of patient vitals, medication administrations, or laboratory tests/results). The definition of a LOT was the first eligible drug episode plus other eligible drugs given within 28 days. The name of the ‘regimen’ for each LOT was the combination of therapies in that line, unless otherwise noted. When a gap in drug episodes of >120 days occurred, the LOT was advanced. For drug substitutions, the line was named according to the drug given for the longest duration within the line. Substitutions in either direction between cisplatin and carboplatin, capecitabine and fluorouracil, or between biosimilars did not advance the LOT. For the most recent LOT, the end date was defined for individuals with no recorded date of death as the date of the last patient-level structured activity (i.e., last record of vitals, medication administration, or laboratory tests or results). For patients with a recorded date of death, the earlier date of the date of death and the last patient-level structured activity was used. For all other lines, the LOT end date was the day before the start date of the next LOT.

### Study outcomes

Real-world OS (rwOS) was defined as the interval in months between index date and the date of death from any cause. Real-world time on treatment (rwToT) was defined as the difference between the first and last dates of administration plus 1 day, expressed in months. Discontinuation of treatment was defined as either initiation of the next line of therapy, patient death while on therapy, or a gap of ≥120 days between the last administration date and last follow-up date (18, 19). If none of the discontinuation criteria were met, patients were considered censored at their last date of treatment administration. Real-world time to next treatment (rwTTNT) was defined as the interval in months between index date and the start of the next line of therapy, or date of death from any cause. Individuals without a record of new treatment or death were censored at data-cutoff.

### Statistical analysis

All study outcomes were summarized separately for the pembrolizumab monotherapy and pembrolizumab plus chemotherapy groups. Descriptive statistics (percentages, means, SDs, medians, ranges, CIs, and interquartile ranges [IQRs]) were used to describe demographic and clinical characteristics. Real-world OS, ToT, and TNT were analyzed using the Kaplan-Meier method with 95% CIs, and summary tables were generated of the number of events and censored patients at different time intervals.

A multivariate stepwise logistic regression model was used to identify baseline patient-level factors associated with initiation of pembrolizumab monotherapy versus pembrolizumab plus chemotherapy in 1L R/M HNSCC. Odds ratios (ORs) and the corresponding 95% CIs were estimated. Cox proportional hazards models using stepwise selection methods were used to simultaneously assess the effects on rwOS of several potential risk factors, including age group, sex, race/ethnicity, smoking status, tumor site, HPV status, Eastern Cooperative Oncology Group performance status (ECOG PS), and CPS for each cohort (pembrolizumab monotherapy and pembrolizumab plus chemotherapy) separately. Cox proportional HRs and the corresponding 95% CIs were estimated. A *p*-value of <0.05 was considered statistically significant in both models. All analyses were conducted in R Studio and SAS.

## Results

### Study population

**Figure 1:**
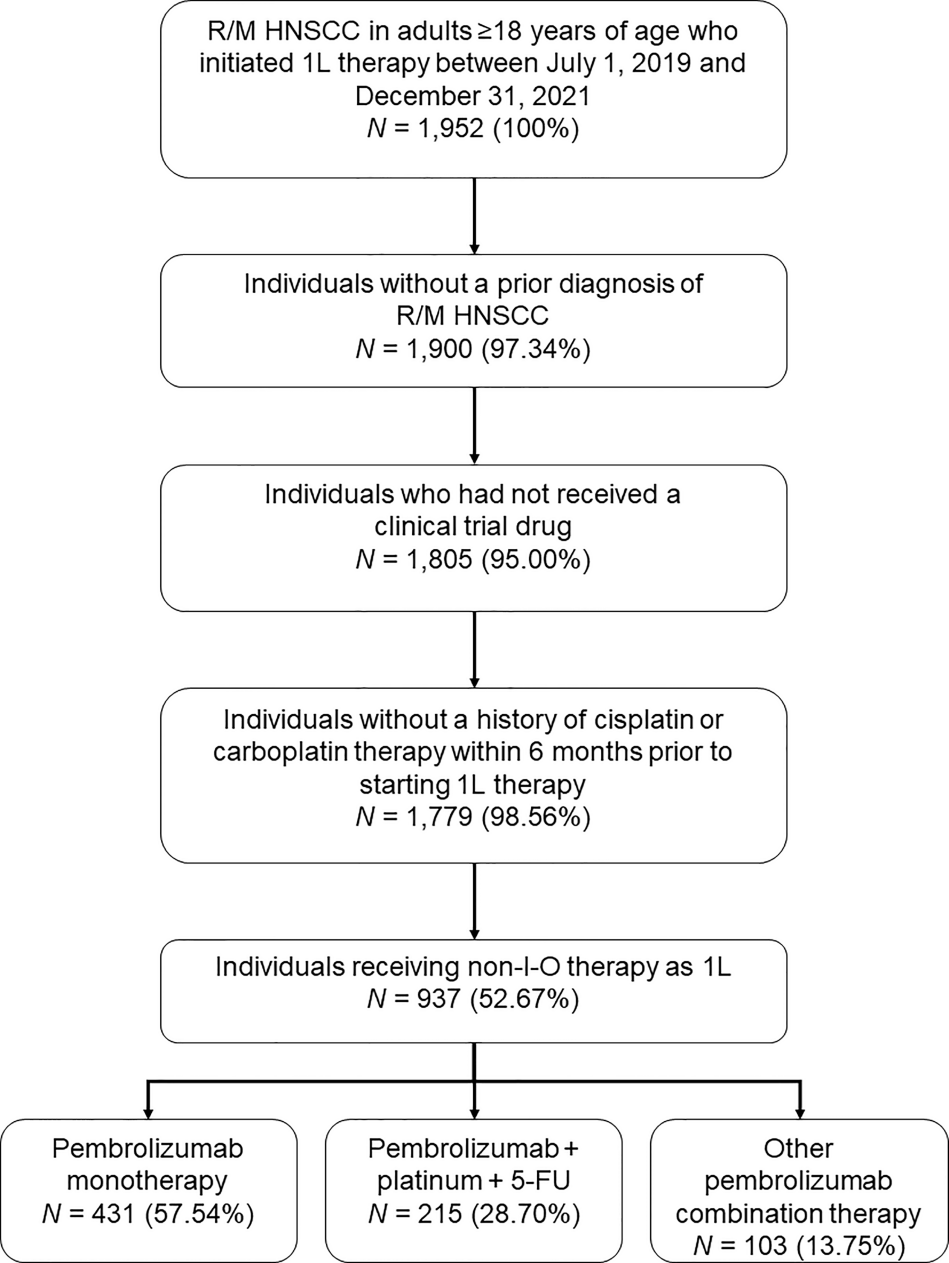
Study population disposition diagram. 1L, first-line; 5-FU, 5-fluorouracil; I-O, immuno-oncology; R/M HNSCC, recurrent or metastatic head and neck squamous cell carcinoma. Percentages are expressed as a percent of the *N* value of the preceding step.

A total of 1,952 adults ≥18 years of age had a diagnosis of R/M HNSCC and initiated 1L therapy between July 1, 2019 and December 31, 2021, of whom 1,779 met all study criteria (**Figure 1**). Among this group, 749 individuals received pembrolizumab-containing therapies, of whom 431 (57.5%) received pembrolizumab monotherapy, 215 (28.7%) received pembrolizumab in combination with platinum and 5-FU (‘pembrolizumab plus chemotherapy’; 84.2% of this group received carboplatin and 15.8% received cisplatin), and 103 (13.8%) received a different pembrolizumab combination therapy. Further analysis of the non-FDA-approved regimens received by the latter group was considered out of scope for the current study.

The baseline demographic and clinical characteristics of all individuals receiving approved 1L pembrolizumab-containing therapies are summarized in **Table 1**. The study population comprised 500 males (77.4%) and 146 females (22.6%) with a median (95% CI) age of 68.0 (66.8–68.3) years. More than half of the study population (59.8%) were White, and most (78.5%) had a history of smoking. The most common tumor site among the study population was the oropharynx (46.0%); 64.6% of oropharyngeal tumors were HPV-positive, compared with 36.1% for all tumor sites. Distant metastatic disease (HNSCC with distant recurrence OR Stage IVc at initial diagnosis) was reported for 56.8% of the study population, HNSCC with locoregional recurrence for 23.1%, HNSCC not cured at initial diagnosis for 16.4%, and HPV-positive Stage IV oropharyngeal tumor at initial diagnosis for 3.7%. The ECOG PS on the index date was 0–1 for 66.1% of the study population, 2–4 for 20.9%, and unknown or not documented for 13.0%. Evidence of PD-L1 testing of any kind was identified for 71.7% of the study population, and a CPS test result was available for 352 individuals (54.5%). A CPS of <1 was recorded for 10.2% of this latter group, while 83.8% had a score of ≥1 and 42.3% had a score of ≥20.

**Table 1:** Baseline demographic and clinical characteristics of individuals receiving 1L pembrolizumab monotherapy or pembrolizumab plus chemotherapy.

Characteristic	1L pembrolizumab (all) (*N* = 646)	1L pembrolizumab monotherapy (*N* = 431)	1L pembrolizumab + chemotherapy (*N* = 215)
Age (years)			
Median (95% CI)	68.0 (66.8–68.3)	69.0 (68.4–70.2)	64.0 (62.8–65.3)
Median (IQR)	68.0 (61.0–65.0)	69.0 (63.0–78.0)	64.0 (59.0–70.0)
Sex			
Male	500 (77.4)	330 (76.6)	170 (79.1)
Race			
White	386 (59.8)	261 (60.6)	125 (58.1)
Black	34 (5.3)	21 (4.9)	13 (6.0)
Asian	6 (0.9)	4 (0.9)	2 (0.9)
Other	220 (34.1)	145 (33.6)	75 (34.9)
Smoking status ^A^			
History of smoking	507 (78.5)	343 (79.6)	164 (76.3)
No history of smoking	138 (21.4)	88 (20.4)	50 (23.3)
Census region			
South	375 (58.0)	251 (58.2)	124 (57.7)
Midwest	78 (12.1)	54 (12.5)	24 (11.2)
West	67 (10.4)	37 (8.6)	30 (14.0)
Northeast	60 (9.3)	40 (9.3)	20 (9.3)
Unknown/not documented	66 (10.2)	49 (11.4)	17 (7.9)
Practice type			
Community	583 (90.2)	384 (89.1)	199 (92.6)
Academic	57 (8.8)	43 (10.0)	14 (6.5)
Both	6 (0.9)	4 (0.9)	2 (0.9)
Primary tumor site(s)			
Oropharynx	297 (46.0)	193 (44.8)	104 (48.4)
Larynx	166 (25.7)	120 (27.8)	46 (21.4)
Oral cavity	138 (21.4)	89 (20.6)	49 (22.8)
Hypopharynx	35 (5.4)	23 (5.3)	12 (5.6)
Other ^B^	10 (1.5)	6 (1.4)	4 (1.9)
Disease status			
Distant metastatic disease (HNSCC with distant recurrence OR Stage IVc at initial diagnosis)	367 (56.8)	238 (55.2)	129 (60.0)
HNSCC with locoregional recurrence ^C^	149 (23.1)	106 (24.6)	43 (20.0)
HNSCC not cured at initial diagnosis	106 (16.4)	77 (17.9)	29 (13.5)
HPV-positive Stage IV oropharyngeal tumor at initial diagnosis	24 (3.7)	10 (2.3)	14 (6.5)
Stage at initial diagnosis			
I–II	115 (17.8)	88 (20.4)	27 (12.6)
III–IVB	326 (50.5)	232 (53.8)	94 (43.7)
IV	24 (3.7)	10 (2.3)	14 (6.5)
IVc	92 (14.2)	41 (9.5)	51 (23.7)
Unknown/not documented	89 (13.8)	60 (13.9)	29 (13.5)
ECOG PS on index date			
0–1	427 (66.1)	265 (61.5)	162 (75.3)
2–4	135 (20.9)	103 (23.9)	32 (14.9)
Unknown/not documented	84 (13.0)	63 (14.6)	21 (9.8)
HPV status (all subtypes)			
Positive	233 (36.1)	163 (37.8)	70 (32.6)
Negative	258 (39.9)	165 (38.3)	93 (43.3)
Equivocal or unknown	155 (24.0)	103 (24.9)	52 (24.2)
HPV status (oropharynx subtype only)	*N* = 297	*N* = 193	*N* = 104
Positive	192 (64.6)	137 (71.0)	55 (52.9)
Negative	87 (29.3)	47 (24.4)	40 (38.5)
Equivocal or unknown	18 (6.1)	9 (4.7)	9 (8.7)
Evidence of PD-L1 testing ^D^			
Yes	463 (71.7)	318 (73.8)	145 (67.4)
CPS ^E^	*N* = 352	*N* = 244	*N* = 108
<1	36 (10.2)	18 (7.4)	18 (16.7)
≥1	295 (83.8)	211 (86.5)	84 (77.8)
≥20	149 (42.3)	109 (44.7)	40 (37.0)
Unknown/not documented	21 (6.0)	15 (6.1)	6 (5.7)

1L, first-line; CI, confidence interval; CPS, combined positive score; ECOG PS, Eastern Cooperative Oncology Group performance status; HNSCC, head and neck squamous cell carcinoma; HPV, human papillomavirus; IQR, interquartile range; PD-L1, programmed death ligand 1.

All values are given as n (%) unless otherwise indicated.

^A^ Smoking status was not documented for 1 individual receiving 1L pembrolizumab + platinum + 5-FU.

^B^ Includes pharynx not otherwise specified, tongue not otherwise specified, or other unspecified tumor site.

^C^ Includes individuals not cured at first locoregional recurrence, or with second locoregional recurrence.

^D^ PD-L1 testing performed at index date ± 30 days.

^E^ If multiple CPS values were available, the score recorded closest to the index date was reported.

### Factors associated with choice of 1L pembrolizumab therapy

A stepwise logistic regression analysis found that pembrolizumab monotherapy was significantly more likely to be used for individuals who were older (*p* < 0.001), had a higher ECOG PS at index date (*p* = 0.004), a tumor located in the larynx (*p* = 0.023), or a higher CPS (*p* = 0.026 for scores of 1–19 versus <1 and *p* = 0.007 for scores of ≥20 versus <1; **Table 2**). Additionally, compared with individuals with HPV-positive Stage IV oropharyngeal tumor at initial diagnosis, individuals with HNSCC not cured at initial diagnosis, distant metastatic disease (HNSCC with distant recurrence OR Stage IVc at initial diagnosis), or HNSCC with locoregional recurrence were more likely to receive pembrolizumab monotherapy (*p* < 0.001 for all comparisons). Pembrolizumab monotherapy was significantly less likely to be used for individuals whose tumors were HPV-negative versus positive (*p* = 0.030) or who were resident in the West census region (*p* = 0.027).

**Table 2:** Factors associated with use of 1L pembrolizumab monotherapy versus pembrolizumab plus chemotherapy: stepwise logistic regression model.

Variable	OR	95% CI	*p*-value
Age (referent: <65 years)			
≥65 years	2.42	1.68–3.52	**<0.001**
Sex (referent: male)			
Female	1.41	0.90–2.22	0.140
ECOG PS on index date (referent: 0–1)			
2–4	2.03	1.26–3.34	**0.004**
Unknown/not defined	2.30	1.29–4.23	**0.006**
History of smoking (referent: no/unknown)			
Yes	1.43	0.89–2.26	0.132
Primary tumor site (referent: oropharynx)			
Hypopharynx	1.46	0.61–3.65	0.403
Larynx	1.84	1.09–3.12	**0.023**
Oral cavity	0.91	0.52–1.59	0.740
Other ^A^	0.99	0.24–4.43	0.998
HPV status (referent: positive)			
Negative	0.59	0.36–0.93	**0.026**
Unknown	0.58	0.32–1.03	0.065
CPS (referent: <1)			
1–19	2.62	1.12–6.12	**0.026**
≥20	3.21	1.37–7.55	**0.007**
Unknown	1.87	0.85–4.09	0.117
Census region (referent: Northeast)			
Midwest	1.31	0.59–2.89	0.495
South	0.88	0.46–1.64	0.704
West	0.40	0.18–0.89	**0.027**
Missing/not defined	1.16	0.50–2.69	0.720
Advanced diagnostic criteria (referent: HPV-positive Stage IV oropharyngeal tumor at initial diagnosis)			
HNSCC not cured at initial diagnosis	4.58	2.49–8.61	**<0.001**
Distant metastatic disease (HNSCC with distant recurrence OR Stage IVc at initial diagnosis)	4.83	2.90–8.17	**<0.001**
HNSCC with locoregional recurrence	4.62	2.61–8.35	**<0.001**

1L, first-line; CI, confidence interval; CPS, combined positive score; ECOG PS, Eastern Cooperative Oncology Group performance status; HNSCC, head and neck squamous cell carcinoma; HPV, human papilloma virus; OR, odds ratio. Statistically significant p-values (<0.05) are presented in bold.

^A^ Includes pharynx not otherwise specified, tongue not otherwise specified, or other unspecified tumor site.

### Real-world outcomes

**Figure 2:**
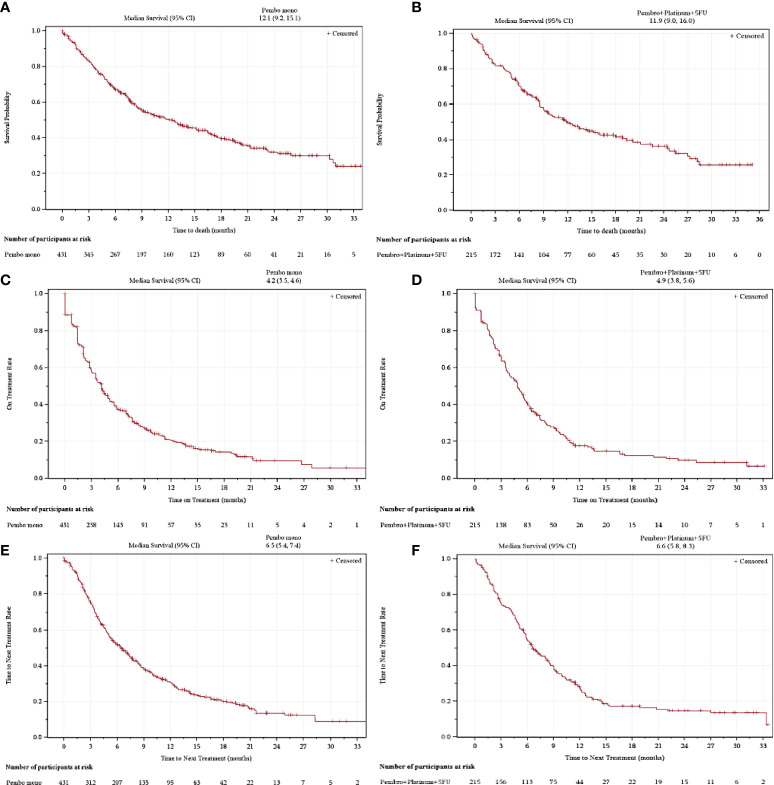
Kaplan-Meier analysis of overall survival **(A, B)**, real-world time on treatment **(C, D)**, and time to next treatment **(E, F)** for individuals receiving 1L pembrolizumab monotherapy (left) or pembrolizumab plus chemotherapy (right).

The median (range) follow-up periods were 7.9 (0.0–35.1) months for individuals who received 1L pembrolizumab monotherapy and 8.5 (0.1–35.1) months for those who received 1L pembrolizumab plus chemotherapy. The median (95% CI) rwOS was 12.1 (9.2–15.1) months among individuals receiving 1L pembrolizumab monotherapy and 11.9 (9.0–16.0) months for those receiving 1L pembrolizumab plus chemotherapy (**Table 3**). The median (95% CI) rwToT for the monotherapy group was 4.2 (3.5–4.6) months, while the median (95% CI) rwTTNT was 6.5 (5.4–7.4) months **Figures 2A, C, E**); the corresponding values for the pembrolizumab plus chemotherapy group were numerically similar, at 4.9 (3.8–5.6) for rwToT and 6.6 (5.8–8.3) months for rwTTNT (**Figures 2B, D, F**).

**Table 3:** Real-world time on treatment, time to next treatment, and overall survival in months among individuals receiving pembrolizumab monotherapy or pembrolizumab plus chemotherapy.

Characteristic	1L pembrolizumab (all) (*N* = 646)	1L pembrolizumab monotherapy (*N* = 431)	1L pembrolizumab + chemotherapy (*N* = 215)
rwToT			
Number of events	516	337	179
Follow-up: median (range)	8.3 (0.0–35.1)	7.9 (0.0–34.3)	8.5 (0.1–35.1)
ToT: median (95% CI)	4.2 (3.7–4.9)	4.2 (3.5–4.6)	4.9 (3.8–5.6)
On treatment rate: % (95% CI)			
At 6 months	38.3 (34.5–42.1)	37.2 (32.5–41.9)	40.5 (33.8–47.1)
At 12 months	19.3 (16.1–22.8)	20.5 (16.4–24.9)	17.6 (12.5–23.4)
At 18 months	13.5 (10.5–16.8)	14.3 (10.6–18.5)	12.3 (7.9–17.9)
At 24 months	9.6 (6.7–13.0)	9.4 (5.7–14.3)	9.8 (5.7–15.2)
rwTTNT			
Number of events	485	317	168
TTNT: median (95% CI)	6.6 (5.8–7.3)	6.5 (5.4–7.4)	6.6 (5.8–8.3)
Yet to initiate 2L treatment: % (95% CI)			
At 6 months	52.6 (48.6–56.6)	52.0 (47.1–56.7)	53.9 (46.9–60.4)
At 12 months	29.6 (25.9–33.4)	30.8 (26.2–35.6)	27.4 (21.3–33.9)
At 18 months	19.0 (15.7–22.6)	20.0 (15.8–24.6)	17.1 (11.9–23.1)
At 24 months	14.3 (11.1–17.9)	13.5 (9.4–18.4)	14.7 (9.8–20.6)
rwOS			
Number of events	367	244	123
OS: median (95% CI)	12.1 (9.8–14.2)	12.1 (9.2–15.1)	11.9 (9.0–16.0)
Survival rate: % (95% CI)			
At 6 months	68.0 (64.2–71.5)	67.0 (62.2–71.3)	70.0 (63.3–75.7)
At 12 months	50.3 (46.1–54.3)	50.6 (45.5–55.5)	49.6 (42.3–56.4)
At 18 months	40.2 (35.9–44.5)	39.6 (34.3–44.8)	41.7 (34.3–48.9)
At 24 months	33.5 (29.0–38.1)	32.0 (26.4–37.7)	36.4 (28.6–44.1)

1L, first-line; 2L, second-line; CI, confidence interval; IQR, interquartile range; OS, overall survival; rw, real-world; ToT, time on treatment; TTNT, time to next treatment.

Values were calculated using the Kaplan-Meier method. All values are in months unless otherwise specified.

### Factors associated with real-world overall survival

A Cox proportional hazards model identified HPV-positive status as a significant predictor of longer rwOS in both treatment groups (*p* = 0.009 for pembrolizumab monotherapy and *p* = 0.028 for pembrolizumab plus chemotherapy; **Table 4**). In addition, among those receiving pembrolizumab monotherapy, lower ECOG PS was identified as a significant predictor of longer rwOS (*p* < 0.001) and tumor site in the oral cavity as a significant predictor of shorter rwOS (*p* = 0.047).

**Table 4:** Factors associated with real-world overall survival: Cox multivariate analysis after stepwise analysis.

Variable	1L pembrolizumab monotherapy group			1L pembrolizumab plus chemotherapy group		
	HR	95% CI	*p*-value	HR	95% CI	*p*-value
ECOG PS on index date (referent: 0–1)						
2–4	2.20	1.64–2.94	**<0.001**	NA	NA	NA
Unknown/not defined	2.00	0.81–1.75	0.354	NA	NA	NA
Primary tumor site (referent: oropharynx)						
Hypopharynx	1.20	0.67–2.12	0.531	NA	NA	NA
Larynx	0.70	0.48–1.02	0.066	NA	NA	NA
Oral cavity	1.49	1.00–2.20	**0.047**	NA	NA	NA
Other ^A^	1.25	0.45–3.44	0.660	NA	NA	NA
HPV status (referent: positive)						
Negative	1.60	1.12–2.26	**0.009**	1.63	1.05–2.54	0.028
Unknown	1.57	1.03–2.39	**0.033**	2.16	1.33–3.51	<0.001
Age (referent: <65 years)						
≥65 years	NA	NA	NA	2.16	1.33–3.51	<0.001

1L, first-line; CI, confidence interval; ECOG PS, Eastern Cooperative Oncology Group performance status; HPV, human papilloma virus; HR, hazard ratio; NA, not applicable.

Statistically significant p-values (<0.05) are presented in bold.

^A^ Includes pharynx not otherwise specified, tongue not otherwise specified, or other unspecified tumor site.

### Real-world overall survival stratified by significant risk factors

Each treatment group was stratified further by the 3 baseline factors that emerged from the Cox model analysis as significant predictors of rwOS (i.e., ECOG PS, tumor site, and tumor HPV status). Among those receiving pembrolizumab monotherapy, the median rwOS was 20 months for those with HPV-positive tumors versus 9 months for those with HPV-negative tumors; 17 months for those with an ECOG PS of 0–1 versus 6 months for those with an ECOG PS of 2–4; and 13 months for those with oropharyngeal tumors versus 9 months for those with tumors of the larynx, 5 months for those with oral cavity tumors, and 6 months for individuals with tumors of the hypopharynx (**Supplementary Figures 1A, C, E**).

Among individuals receiving pembrolizumab plus chemotherapy, the median rwOS was 15 months for those with HPV-positive tumors versus 7 months for those with HPV-negative tumors; 13 months for those with an ECOG PS of 0–1 versus 11 months for those with an ECOG PS of 2–4; and 18 months for those with oropharyngeal tumors versus 10 months for those with tumors of the larynx, 11 months for those with oral cavity tumors, and 9 months for individuals with tumors of the hypopharynx (**Supplementary Figures 1B, D, F**).

### Second-line treatments

**Figure 3:**
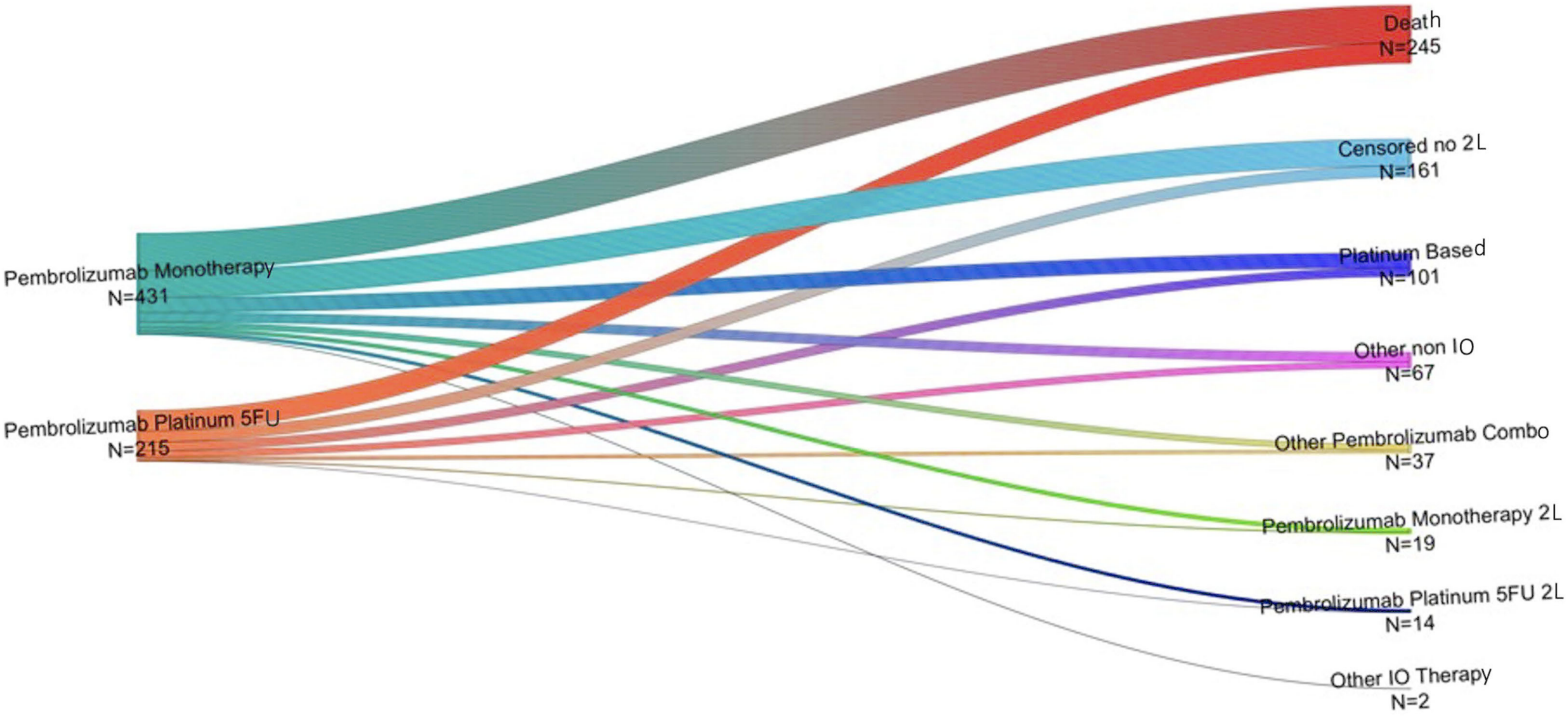
Second-line therapies (right) received by individuals who received pembrolizumab monotherapy or pembrolizumab + chemotherapy (left) as 1L treatment for R/M HNSCC*. L1, first-line therapy; L2, second-line therapy; LOT, line of treatment. *Other IO includes patients receiving nivolumab, Ipilimumab, Atezolizumab and Cemiplimab monotherapy or in combination with chemotherapy. Other non-IO includes patients receiving chemotherapy (mono or in combination) and taxanes monotherapy.

One hundred fifty-eight patients received pembrolizumab monotherapy as 1L treatment for R/M HNSCC and subsequently received a 2L treatment during the study period. The most common 2L treatment after pembrolizumab monotherapy was a platinum-based regimen (40.5%), followed by another non-IO regimen (25.3%; **Figure 3**). Second-line pembrolizumab + chemotherapy was received by 8.2% of this group and other 2L pembrolizumab combination therapies by 15.2%. Eighty-two patients received pembrolizumab + chemotherapy as 1L treatment and also received a 2L therapy during the study period. The most common 2L treatments in this group were platinum-based and other non-IO regimens (45.1% and 32.9%, respectively); 5% of this group received 2L pembrolizumab monotherapy and 15.8% received another 2L pembrolizumab combination therapy.

## Discussion

In this study we described the demographic and clinical characteristics of individuals receiving FDA-approved 1L pembrolizumab-containing therapies for R/M HNSCC in real-word US settings, identified factors associated with use of pembrolizumab monotherapy versus pembrolizumab plus chemotherapy, and estimated the median rwOS, rwToT, and rwTTNT for individuals receiving these treatments. We also identified factors associated with rwOS outcomes with 1L pembrolizumab treatment. To our knowledge, this is the largest study to date of real-world outcomes of 1L pembrolizumab-containing therapies for R/M HNSCC in the US. The study adds to the findings from the registered clinical trial (KEYNOTE-048) and from previous smaller real-world studies conducted in the US and Japan (11, 20, 21). The median OS outcomes from the current study were numerically similar to those of KEYNOTE-048.

Participants in randomized controlled trials (RCTs) often differ demographically and clinically from the population who ultimately receive approved treatments in real-world settings (22). As with an estimated 60% of oncology RCTs, only individuals with an ECOG PS of 0–1 were included in KEYNOTE-048 (11, 22); in contrast, 21% of the current study population had an ECOG PS of 2–4, indicating higher levels of functional impairment and poorer overall health status. This finding reflects the high individual burden of disease in the current study population. The current study population was also numerically older than the KEYNOTE-048 population (median age 69 versus 62 years for those receiving pembrolizumab monotherapy and 64 versus 61 years for those receiving pembrolizumab plus chemotherapy), but had a similar proportion of males and of individuals with a history of smoking (~80% in all cases) (11). Similar demographics have been observed in other R/M HNSCC observational studies and clinical trials (19, 21, 23–27). The proportions of tumors with CPS ≥1 and ≥20 were similar between the current and KEYNOTE-048 populations, although CPS values were not available for almost half of the current study population. This low testing rate may be related to the fact that >90% of the study population were treated at community rather than academic centers. Human papillomavirus (HPV) status was not assessed directly in KEYNOTE-048, but p16 status was used as a surrogate for individuals with oropharyngeal tumors: 21–22% of this group of KEYNOTE-048 participants had p16-positive tumors (11). In the current study, among individuals with a recorded unequivocal HPV status, the proportion of HPV-positive tumors was higher at 69% (oropharynx subtype) and 47% (all subtypes), in line with published reports (21, 28). The KEYNOTE-048 population also included more individuals with tumors in the hypopharynx than did the current study population (13–16% versus 5%) (11). Stage was not reported in KEYNOTE-048, and locoregional and distant recurrence were not distinguished (11).

Despite these differences, the median rwOS values observed in the current study were similar to the median OS values reported in KEYNOTE-048 (12.1 versus 11.6 months for pembrolizumab monotherapy and 11.9 versus 13.0 months for pembrolizumab plus chemotherapy) (11). A recent US real-world study of 65 individuals receiving first-line pembrolizumab monotherapy for R/M HNSCC in a single academic center reported a median OS of 8.8 months, which was not statistically significantly different from the KEYNOTE-048 trial, although the authors concluded that the study was likely underpowered for this comparison (21). In KEYNOTE-048, the 12- and 24-month OS rates were, respectively, 49% and 29% for the pembrolizumab monotherapy group and 53% and 29% for the pembrolizumab chemotherapy group, which is similar to the corresponding values of 51% and 32% for pembrolizumab monotherapy and 50% and 36% for pembrolizumab plus chemotherapy in the current study (11). A recent real-world study of individuals receiving pembrolizumab-containing therapies in Japan with similar overall baseline demographic and clinical characteristics to the current study population found a similar 1-year OS rate for pembrolizumab monotherapy (52%, *n* = 60), but a higher 1-year OS rate of 73% (*n* = 37) for those receiving pembrolizumab plus chemotherapy (20). The latter result may reflect the small sample size and/or other differences from the current study population, motivating further ongoing study of real-world outcomes with pembrolizumab-containing 1L therapies for R/M HNSCC in diverse populations.

The median rwToTs observed in the current study were also numerically similar to the corresponding median durations of study therapy observed in KEYNOTE-048 (4.2 versus 3.5 months for pembrolizumab monotherapy and 4.9 versus 5.8 months for pembrolizumab plus chemotherapy) (11). The maximum treatment period during KEYNOTE-048 was 2 years; at the 2-year timepoint in the current study, 9% of those receiving pembrolizumab monotherapy and 10% of those receiving pembrolizumab plus chemotherapy were still on treatment. The median progression-free survival (PFS) in KEYNOTE-048 was 2.3 months for the pembrolizumab monotherapy group and 4.9 months for the pembrolizumab plus chemotherapy group (11). The current study used rwTTNT as a proxy for PFS, an approach that has been validated in similar oncology drug study settings (29, 30). The median rwTTNTs were numerically longer than the PFS values from KEYNOTE-048, at 6.5 months for the pembrolizumab monotherapy group and 6.6 months for the pembrolizumab chemotherapy group. Delays between detection of a progression event and initiation of the next LOT may account for some of this difference.

In the current study, median rwOS was numerically similar between the pembrolizumab plus chemotherapy and the pembrolizumab monotherapy groups. As expected, positive HPV status was significantly associated with longer rwOS in both treatment groups; in the pembrolizumab monotherapy group, lower ECOG PS was significantly associated with longer rwOS and tumor site in the oral cavity with shorter rwOS. Further analysis of rwOS stratified by these 3 baseline variables confirmed their importance as predictors of rwOS in the respective group(s). Our findings are consistent with those of a previous small study of individuals receiving pembrolizumab and/or nivolumab to treat R/M HNSCC, which reported a median OS of 15.1 months for individuals with p16^+^ oropharyngeal disease versus 4.5 months for those with p16^–^ oropharyngeal disease (31). In contrast, a recent US real-world study of first-line pembrolizumab monotherapy for R/M HNSCC did not find an association between p16 status and OS (21). Nevertheless, the association between HPV status and clinical outcomes in HNSCC is well established (and has been reflected in the American Joint Committee on Cancer’s staging guidelines since 2017): HPV-negative status is associated with higher tumor mutational burden, shorter OS, higher risk of progression, and lower rate of response to chemotherapy and radiation treatments (28, 32–34). In addition, metastatic HPV-positive HNSCC often presents as low tumor-burden lung nodules with a more favorable prognosis compared to other forms of metastatic HNSCC. Higher ECOG PS has also been found to correlate with significantly shorter OS in individuals receiving pembrolizumab monotherapy, other immune checkpoint inhibitors, and other treatments for R/M HNSCC and in individuals receiving pembrolizumab monotherapy for advanced non-small cell lung cancer, as well as with a higher rate of adverse events during cancer treatment and of treatment discontinuation due to death (21, 35–41). Shorter OS for oral tumors than for other HNSCC tumor sites has not generally been reported in the curative setting for R/M HNSCC (42), and this finding thus warrants further investigation of the interplay between tumor site and other baseline individual characteristics such as CPS in this treatment group.

Although the 1L pembrolizumab monotherapy and pembrolizumab plus chemotherapy groups had similar clinical outcomes, their baseline characteristics differed. For example, higher baseline CPS was identified as a factor associated with the use of 1L pembrolizumab monotherapy rather than pembrolizumab plus chemotherapy. This pattern is consistent with the results of KEYNOTE-048, the FDA approval, and current clinical guidelines (10, 11, 14). Evidence of PD-L1 expression testing of any kind was observed for 72% of the overall study population (74% of the pembrolizumab monotherapy group and 67% of the pembrolizumab plus chemotherapy group), and CPS values were available for 55% of the study population (57% for the pembrolizumab monotherapy group and 50% of the pembrolizumab plus chemotherapy group). A previous real-world analysis of first-line pembrolizumab monotherapy for R/M HNSCC also reported that the CPS was unknown for almost half (43.7%) of the study participants (21). The intentions of treating physicians might drive treatment choices even in the absence of CPS testing; nevertheless, increased rates of CPS testing in real-world settings may be needed to help guide 1L pembrolizumab treatment for R/M HNSCC.

We also found that use of 1L pembrolizumab monotherapy rather than pembrolizumab plus chemotherapy was associated with HPV-positive tumor status, older age, higher ECOG PS, and laryngeal tumor site. These findings may reflect the selection of the more aggressive combination treatment for some individuals with a worse prognosis, such as those with HPV-negative versus HPV-positive tumor status (32, 33). The selection of pembrolizumab monotherapy for individuals who are older and/or sicker (i.e., with a higher ECOG PS, having smoking-related laryngeal disease, experiencing nutritional compromise caused by swallowing difficulties associated with laryngeal cancer, or being in recovery from recent invasive treatments such as laryngectomies) may also play a role, as this population may be deemed less likely to be able to tolerate the greater side-effects of the more aggressive pembrolizumab plus chemotherapy option (11).

For both treatment groups, the most common 2L treatments received during the study period were platinum-based and other non-IO regimens. A much smaller proportion of each group switched from pembrolizumab monotherapy to pembrolizumab plus chemotherapy, or vice versa. The relatively small proportion of individuals receiving a 1L pembrolizumab-containing therapy who subsequently received another PD-1/PD-L1 inhibitor-containing 2L therapy is consistent with findings from KEYNOTE-048 (11, 13). The most recent update from the trial reported that 3 of 11 individuals receiving second-course pembrolizumab had an objective response, suggesting that this may be a viable option for some patients (13); however, larger studies are needed to confirm this preliminary finding and to identify factors associated with successful 2L treatment following 1L treatment with pembrolizumab-containing therapies (13).

This study extracted clinical and demographic measures from the largest available US source of real-world longitudinal oncology data and thus has a relatively large sample size. The baseline demographic and clinical characteristics of the study population were broadly similar to those reported in previous clinical trials and observational studies of R/M HNSCC, although the current study population had a numerically higher median age and higher proportion of HPV-positive tumors compared to the KEYNOTE-048 population (11, 19, 21, 23–27). The current study also included a more heterogeneous population than did KEYNOTE-048. Most notably, it included individuals with an ECOG PS of 2–4 at the time of treatment initiation, who account for a substantial proportion of the overall R/M HNSCC population (21% of the current study population) but were not eligible for the trial. The findings of the current study are therefore likely to have strong generalizability in real-world settings.

Study limitations are also noted. Although we used the largest available dataset, documentation and coding biases exist, and the baseline demographic and clinical characteristics of the study population may differ from those of all individuals diagnosed with R/M HNSCC in the US. For example, >90% of the study population were treated in community oncology practices, which may be associated with different patient demographics or treatment patterns compared with other practice types. The predominance of this treatment location within the study population may thus introduce bias. The higher proportion of HPV-positive tumors in the current study compared to the KEYNOTE-048 clinical trial may also have affected our survival outcome and other analyses, and limits our ability to directly compare outcomes between these populations. Combined positive score test results were unavailable for many members of the study population, precluding an analysis of real-world outcomes stratified by CPS, as was performed in KEYNOTE-048 (11). This low testing rate, which may reflect current real-world practice, also limits our ability to directly compare the real-world survival outcomes of the current study population with those of the KEYNOTE-048 trial participants and other real-world populations. Finally, no information was available on response rates, toxicity, or the reasons for therapy selection and discontinuation, and we were therefore unable to assess factors that might have affected our findings such as specific adverse events or general tolerability. The lack of information on the rationale for clinical decision making also precludes us from assessing why only 42% of the study population received a pembrolizumab-containing therapy and why no CPS value was available for almost half of the population.

In conclusion, this study provides information on the use of pembrolizumab-containing therapies for HNSCC in real-world US settings. OS outcomes were comparable between the pembrolizumab monotherapy and pembrolizumab plus chemotherapy groups. The survival outcomes of both groups were also similar to the corresponding data observed in a registration clinical trial, despite the real-world study population being more heterogeneous. These findings may help guide the future implementation of pembrolizumab-containing therapies as standard of care 1L treatment for R/M HNSCC.

## Data availability statement

The original contributions presented in the study are included in the article/**Supplementary Material**. Further inquiries can be directed to the corresponding author.

## Ethics statement

Ethical review and approval was not required for the study on human participants in accordance with the local legislation and institutional requirements. Written informed consent for participation was not required for this study in accordance with the national legislation and the institutional requirements.

## Author contributions

CB, GJH, LW, KR, DG, VT, and GMH contributed to the conception and design of the study. DG, VT, and GMH performed and statistical analyses and analyzed the data. All authors interpreted the results. All authors contributed to manuscript revisions, read, and approved the submission. All authors contributed to the article and approved the submitted version.
