# Corrigendum: Real-world treatment patterns and outcomes among individuals receiving first-line pembrolizumab therapy for recurrent/metastatic head and neck squamous cell carcinoma

**DOI:** 10.3389/fonc.2023.1240947

**Published:** 2023-07-17

**Authors:** Christopher M. Black, Glenn J. Hanna, Liya Wang, Karthik Ramakrishnan, Daisuke Goto, Vladimir Turzhitsky, Gleicy M. Hair

**Affiliations:** ^1^ Center for Observational and Real-World Evidence (CORE), Merck & Co., Inc., Rahway, NJ, United States; ^2^ Center for Head & Neck Oncology, Dana-Farber Cancer Institute, Boston, MA, United States

**Keywords:** head and neck squamous cell carcinoma, antineoplastic agents, immunological, antibodies, Kaplan-Meier estimate, patient outcomes, real-world observational study, treatment patterns

**Error in Figure/Table**

In the published article, there was an error in **Table 1** as published. The mean (SD) for the 1L pembrolizumab + chemotherapy column for CPS<1 should be 18 (16.7), not 18 (6.7) as originally shown. Further, we used the terminology, CPS<1% in the first column and the correct terminology is CPS<1. The corrected **Table 1** and its caption appear below.

**Table 1:** Baseline demographic and clinical characteristics of individuals receiving 1L pembrolizumab monotherapy or pembrolizumab plus chemotherapy.

Characteristic	1L pembrolizumab (all) (*N* = 646)	1L pembrolizumab monotherapy (*N* = 431)	1L pembrolizumab + chemotherapy (*N* = 215)
Age (years)			
Median (95% CI)	68.0 (66.8–68.3)	69.0 (68.4–70.2)	64.0 (62.8–65.3)
Median (IQR)	68.0 (61.0–65.0)	69.0 (63.0–78.0)	64.0 (59.0–70.0)
Sex			
Male	500 (77.4)	330 (76.6)	170 (79.1)
Race			
White	386 (59.8)	261 (60.6)	125 (58.1)
Black	34 (5.3)	21 (4.9)	13 (6.0)
Asian	6 (0.9)	4 (0.9)	2 (0.9)
Other	220 (34.1)	145 (33.6)	75 (34.9)
Smoking status ^A^			
History of smoking	507 (78.5)	343 (79.6)	164 (76.3)
No history of smoking	138 (21.4)	88 (20.4)	50 (23.3)
Census region			
South	375 (58.0)	251 (58.2)	124 (57.7)
Midwest	78 (12.1)	54 (12.5)	24 (11.2)
West	67 (10.4)	37 (8.6)	30 (14.0)
Northeast	60 (9.3)	40 (9.3)	20 (9.3)
Unknown/not documented	66 (10.2)	49 (11.4)	17 (7.9)
Practice type			
Community	583 (90.2)	384 (89.1)	199 (92.6)
Academic	57 (8.8)	43 (10.0)	14 (6.5)
Both	6 (0.9)	4 (0.9)	2 (0.9)
Primary tumor site(s)			
Oropharynx	297 (46.0)	193 (44.8)	104 (48.4)
Larynx	166 (25.7)	120 (27.8)	46 (21.4)
Oral cavity	138 (21.4)	89 (20.6)	49 (22.8)
Hypopharynx	35 (5.4)	23 (5.3)	12 (5.6)
Other ^B^	10 (1.5)	6 (1.4)	4 (1.9)
Disease status			
Distant metastatic disease (HNSCC with distant recurrence OR Stage IVc at initial diagnosis)	367 (56.8)	238 (55.2)	129 (60.0)
HNSCC with locoregional recurrence ^C^	149 (23.1)	106 (24.6)	43 (20.0)
HNSCC not cured at initial diagnosis	106 (16.4)	77 (17.9)	29 (13.5)
HPV-positive Stage IV oropharyngeal tumor at initial diagnosis	24 (3.7)	10 (2.3)	14 (6.5)
Stage at initial diagnosis			
I–II	115 (17.8)	88 (20.4)	27 (12.6)
III–IVB	326 (50.5)	232 (53.8)	94 (43.7)
IV	24 (3.7)	10 (2.3)	14 (6.5)
IVc	92 (14.2)	41 (9.5)	51 (23.7)
Unknown/not documented	89 (13.8)	60 (13.9)	29 (13.5)
ECOG PS on index date			
0–1	427 (66.1)	265 (61.5)	162 (75.3)
2–4	135 (20.9)	103 (23.9)	32 (14.9)
Unknown/not documented	84 (13.0)	63 (14.6)	21 (9.8)
HPV status (all subtypes)			
Positive	233 (36.1)	163 (37.8)	70 (32.6)
Negative	258 (39.9)	165 (38.3)	93 (43.3)
Equivocal or unknown	155 (24.0)	103 (24.9)	52 (24.2)
HPV status (oropharynx subtype only)	*N* = 297	*N* = 193	*N* = 104
Positive	192 (64.6)	137 (71.0)	55 (52.9)
Negative	87 (29.3)	47 (24.4)	40 (38.5)
Equivocal or unknown	18 (6.1)	9 (4.7)	9 (8.7)
Evidence of PD-L1 testing ^D^			
Yes	463 (71.7)	318 (73.8)	145 (67.4)
CPS ^E^	*N* = 352	*N* = 244	*N* = 108
<1	36 (10.2)	18 (7.4)	18 (16.7)
≥1	295 (83.8)	211 (86.5)	84 (77.8)
≥20	149 (42.3)	109 (44.7)	40 (37.0)
Unknown/not documented	21 (6.0)	15 (6.1)	6 (5.7)

1L, first-line; CI, confidence interval; CPS, combined positive score; ECOG PS, Eastern Cooperative Oncology Group performance status; HNSCC, head and neck squamous cell carcinoma; HPV, human papillomavirus; IQR, interquartile range; PD-L1, programmed death ligand 1.

All values are given as n (%) unless otherwise indicated.

^A^ Smoking status was not documented for 1 individual receiving 1L pembrolizumab + platinum + 5-FU.

^B^ Includes pharynx not otherwise specified, tongue not otherwise specified, or other unspecified tumor site.

^C^ Includes individuals not cured at first locoregional recurrence, or with second locoregional recurrence.

^D^ PD-L1 testing performed at index date ± 30 days.

^E^ If multiple CPS values were available, the score recorded closest to the index date was reported.

In the published article, there was an error in **Table 2** as published. We used the terminology CPS (referent: <1%) in column one and the correct terminology is CPS (referent: <1). The corrected **Table 2** and its caption appear below.

**Table 2:** Factors associated with use of 1L pembrolizumab monotherapy versus pembrolizumab plus chemotherapy: stepwise logistic regression model.

Variable	OR	95% CI	*p*-value
Age (referent: <65 years)			
≥65 years	2.42	1.68–3.52	**<0.001**
Sex (referent: male)			
Female	1.41	0.90–2.22	0.140
ECOG PS on index date (referent: 0–1)			
2–4	2.03	1.26–3.34	**0.004**
Unknown/not defined	2.30	1.29–4.23	**0.006**
History of smoking (referent: no/unknown)			
Yes	1.43	0.89–2.26	0.132
Primary tumor site (referent: oropharynx)			
Hypopharynx	1.46	0.61–3.65	0.403
Larynx	1.84	1.09–3.12	**0.023**
Oral cavity	0.91	0.52–1.59	0.740
Other ^A^	0.99	0.24–4.43	0.998
HPV status (referent: positive)			
Negative	0.59	0.36–0.93	**0.026**
Unknown	0.58	0.32–1.03	0.065
CPS (referent: <1)			
1–19	2.62	1.12–6.12	**0.026**
≥20	3.21	1.37–7.55	**0.007**
Unknown	1.87	0.85–4.09	0.117
Census region (referent: Northeast)			
Midwest	1.31	0.59–2.89	0.495
South	0.88	0.46–1.64	0.704
West	0.40	0.18–0.89	**0.027**
Missing/not defined	1.16	0.50–2.69	0.720
Advanced diagnostic criteria (referent: HPV-positive Stage IV oropharyngeal tumor at initial diagnosis)			
HNSCC not cured at initial diagnosis	4.58	2.49–8.61	**<0.001**
Distant metastatic disease (HNSCC with distant recurrence OR Stage IVc at initial diagnosis)	4.83	2.90–8.17	**<0.001**
HNSCC with locoregional recurrence	4.62	2.61–8.35	**<0.001**

1L, first-line; CI, confidence interval; CPS, combined positive score; ECOG PS, Eastern Cooperative Oncology Group performance status; HNSCC, head and neck squamous cell carcinoma; HPV, human papilloma virus; OR, odds ratio. Statistically significant p-values (<0.05) are presented in bold.

^A^ Includes pharynx not otherwise specified, tongue not otherwise specified, or other unspecified tumor site.

**Text Correction**

In the published article, there was an error to the text in **Results**, *Study population*, where "CPS<1" was referred to as "CPS<1%".

This sentence previously stated: “A CPS of <1% was recorded for 10.2% of this latter group, while 83.8% had a score of ≥1 and 42.3% had a score of ≥20.”

The corrected sentence appears below:

“A CPS of <1 was recorded for 10.2% of this latter group, while 83.8% had a score of ≥1 and 42.3% had a score of ≥20.”

The authors apologize for these errors and state that these does not change the scientific conclusions of the article in any way. The original article has been updated.

