# Supplementary material for: Real-world treatment patterns and outcomes among individuals receiving first-line pembrolizumab therapy for recurrent/metastatic head and neck squamous cell carcinoma
Source: Front Oncol. 2023 May 22;13:1160144. doi: 10.3389/fonc.2023.1160144 (PMC10241070; doi:10.3389/fonc.2023.1160144)
Supplement: Supplementary file 1 [file DataSheet_1.docx]

## Supplementary Materials

Supplementary Table 1. International Classification of Diseases codes used to identify head and neck cancers

| Category | ICD-9 | ICD-10 |
| --- | --- | --- |
| Inclusion criteria | 140x, 141x, 143x, 144x, 145x, 146x, 147x, 148x, 149x, 161x | C00x, C01x, C02x, C03x, C04x, C05x, C06x, C09x, C10x, C11x, C12x, C13x, C14x, C32x |
| Exclusion criteria | 140.x, 142.x, 147.x, 149.x, 160.0, 160.2, 160.3, 160.4,160.5, 160.8, 160.9 | NA |

ICD, International Classification of Diseases; NA, not applicable.

Supplementary Figure 1. Kaplan-Meier analysis of real-world overall survival stratified by ECOG performance status (top), HPV status (middle), and tumor site (bottom) for individuals receiving 1L pembrolizumab monotherapy (left) or pembrolizumab plus chemotherapy (right).

B)

D)

C)

E)

F)


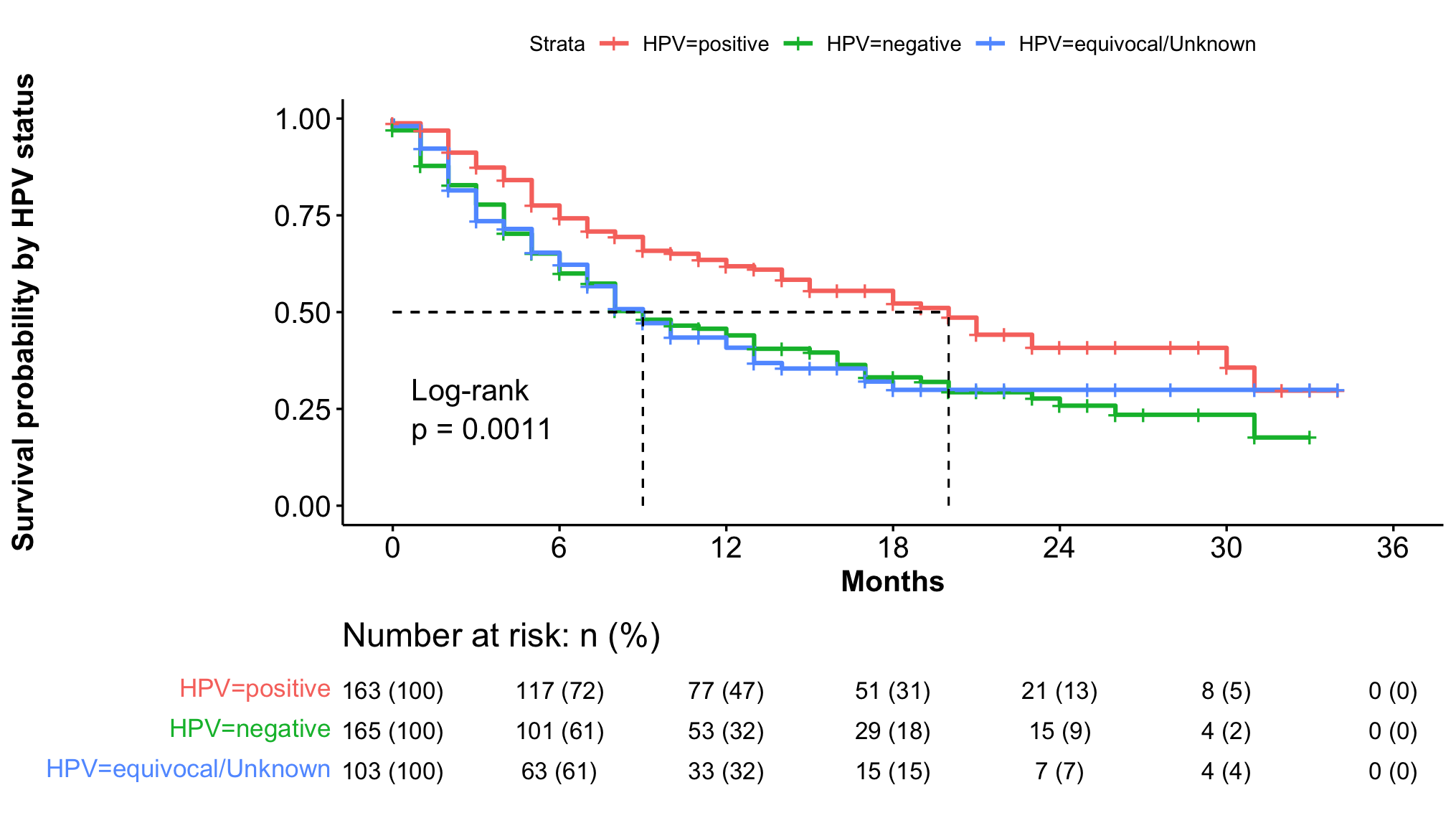

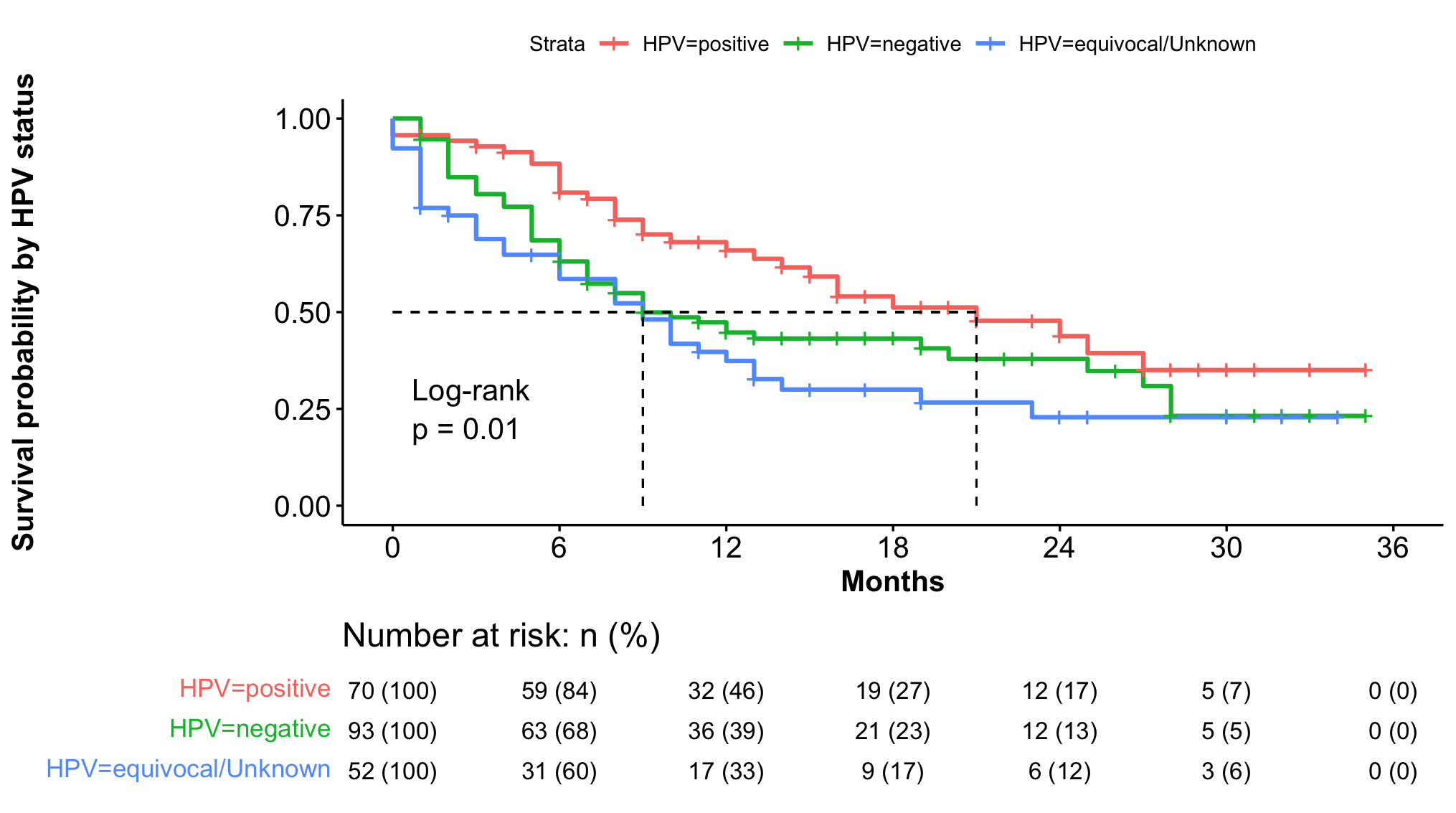

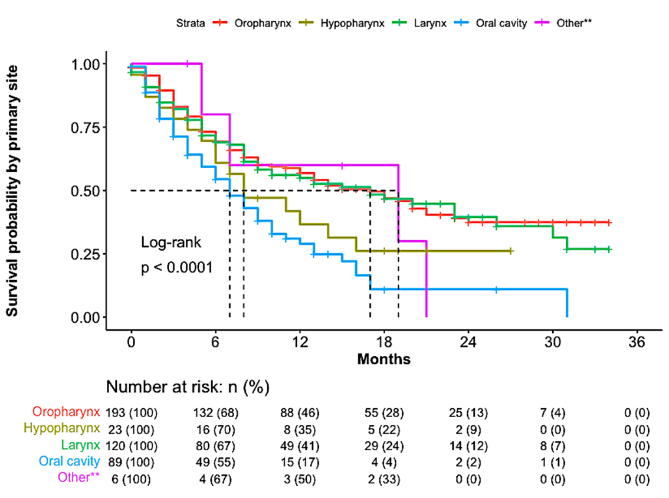

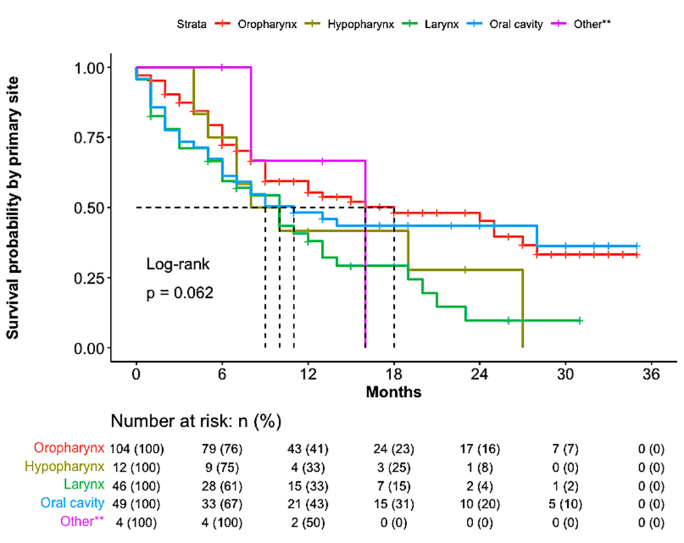

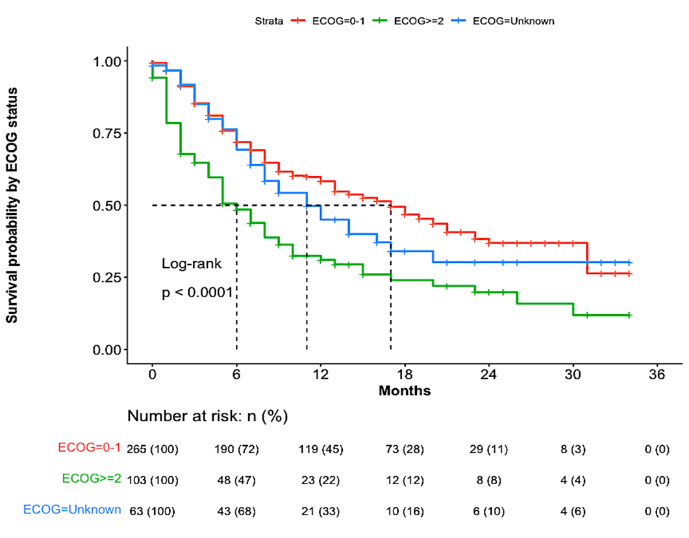

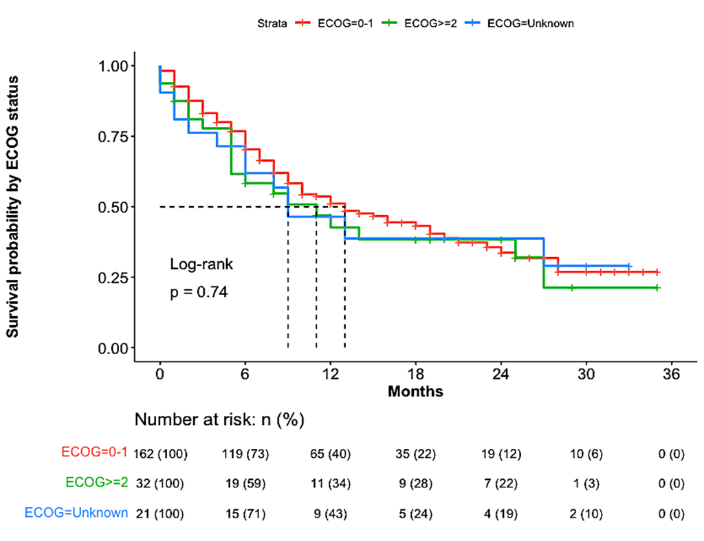


A)

ECOG, Eastern Cooperative Oncology Group; HPV, human papillomavirus.
